# Supplementary material for: Gene-Based Association Tests Using New Polygenic Risk Scores and Incorporating Gene Expression Data
Source: Genes (Basel). 2022 Jun 22;13(7):1120. doi: 10.3390/genes13071120 (PMC9318573; doi:10.3390/genes13071120)

Supplementary Information

Figure S1. Powers of the seven tests versus the total effect size β for a quantitative trait with $N = 5,000$. The proportion of causal variants is 0.3. Models 1-3 corresponding to Genes 1-3 where we only use the eQTL with the largest weight to generate gene expression; Models 4-6 corresponding to Genes 1-3 where we use two eQTLs with the first two largest weights to generate gene expression.

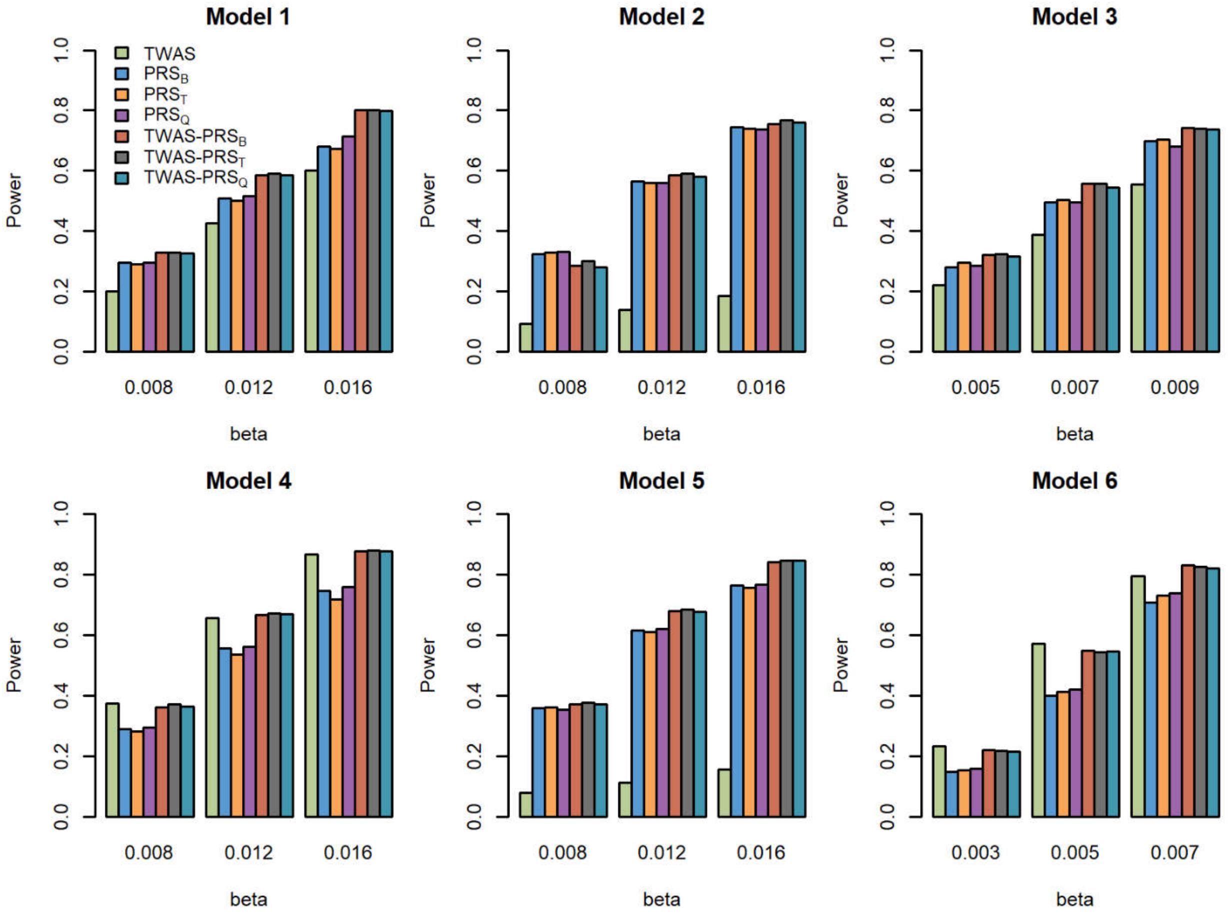

Figure S2. Powers of the seven tests versus the total effect size β for a quantitative trait with $N = 10,000$. The proportion of causal variants is 0.3. Models 1-3 corresponding to Genes 1-3 where we only use the eQTL with the largest weight to generate gene expression; Models 4-6 corresponding to Genes 1-3 where we use two eQTLs with the first two largest weights to generate gene expression.

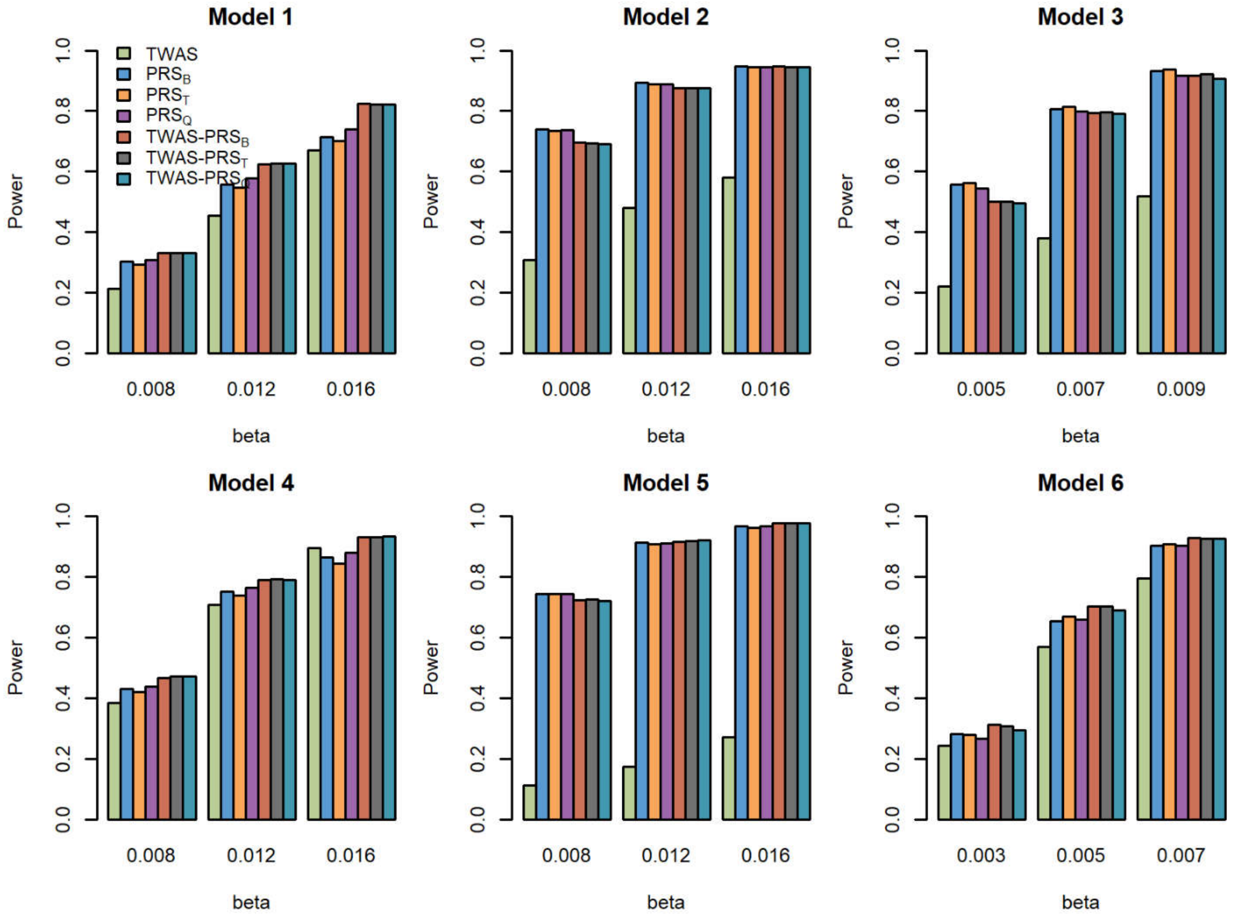

Figure S3. Powers of the seven tests versus the total effect size β for a quantitative trait with $N = 20,000$. The proportion of causal variants is 0.3. Models 1-3 corresponding to Genes 1-3 where we only use the eQTL with the largest weight to generate gene expression; Models 4-6 corresponding to Genes 1-3 where we use two eQTLs with the first two largest weights to generate gene expression.

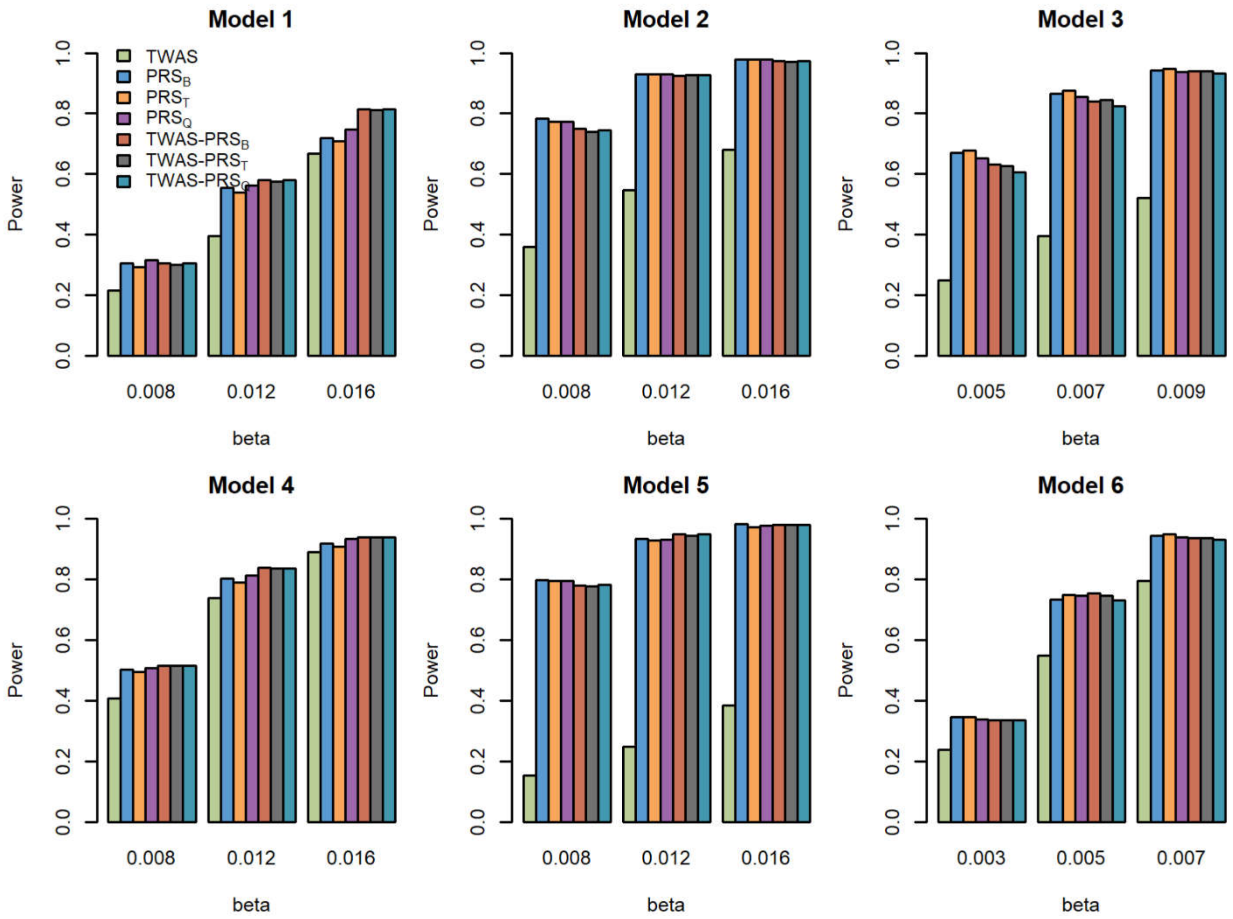

Figure S4. Powers of the seven tests versus the total effect size β for a qualitative trait with $N = 5,000$. The proportion of causal variants is 0.2. Models 1-3 corresponding to Genes 1-3 where we only use the eQTL with the largest weight to generate gene expression; Models 4-6 corresponding to Genes 1-3 where we use two eQTLs with the first two largest weights to generate gene expression.

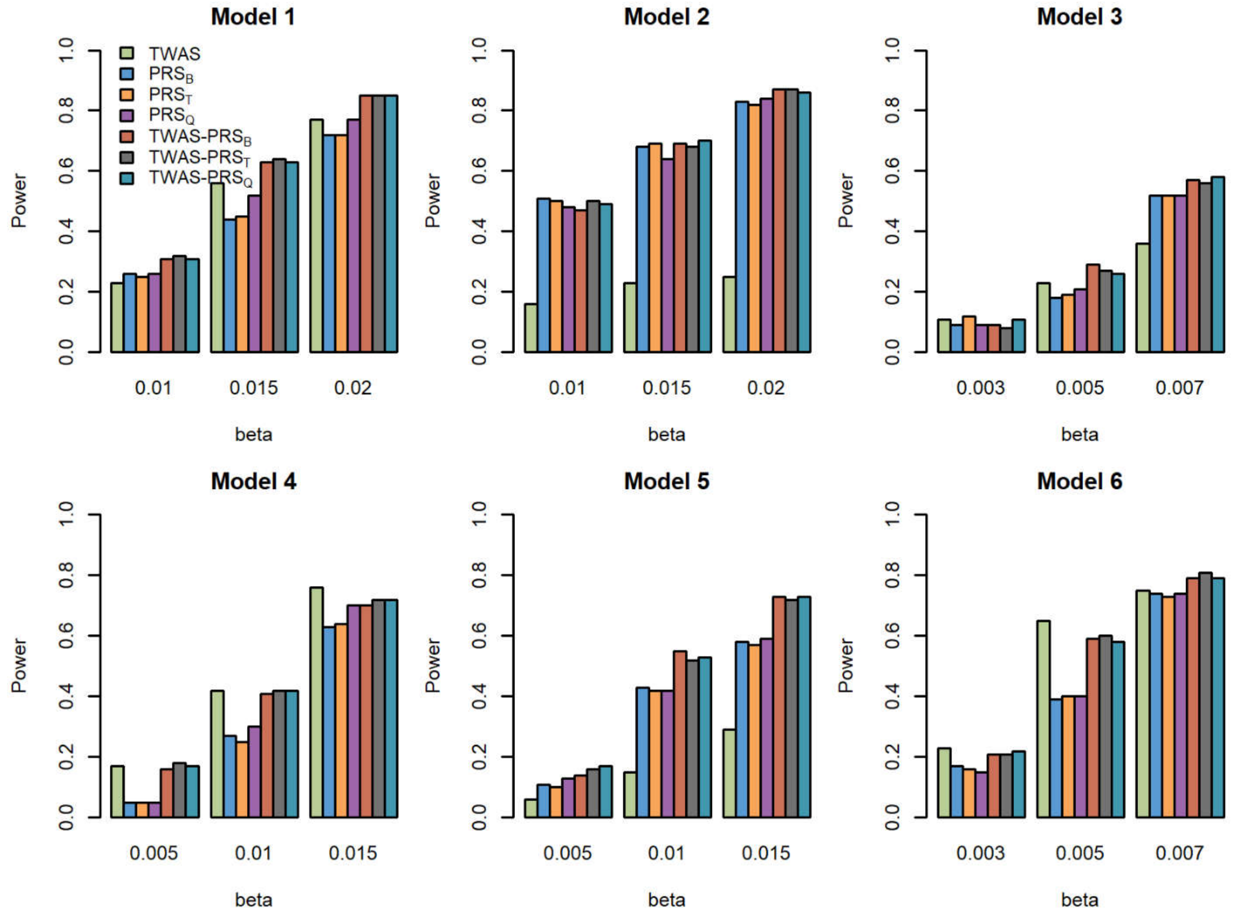

Figure S5. Powers of the seven tests versus the total effect size β for a qualitative trait with $N = 10,000$. The proportion of causal variants is 0.2. Models 1-3 corresponding to Genes 1-3 where we only use the eQTL with the largest weight to generate gene expression; Models 4-6 corresponding to Genes 1-3 where we use two eQTLs with the first two largest weights to generate gene expression.

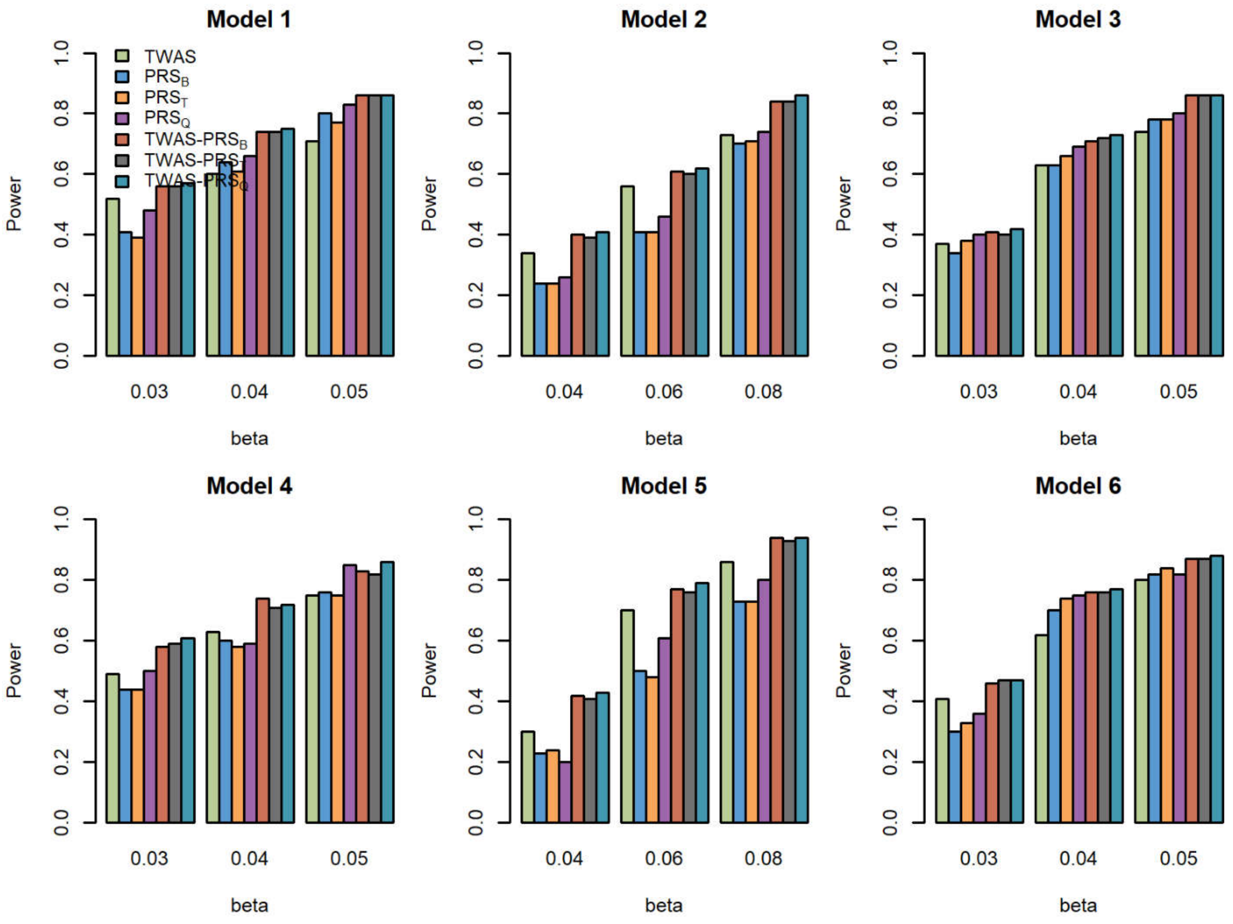

Figure S6. Powers of the seven tests versus the total effect size β for a qualitative trait with $N = 20,000$. The proportion of causal variants is 0.2. Models 1-3 corresponding to Genes 1-3 where we only use the eQTL with the largest weight to generate gene expression; Models 4-6 corresponding to Genes 1-3 where we use two eQTLs with the first two largest weights to generate gene expression.

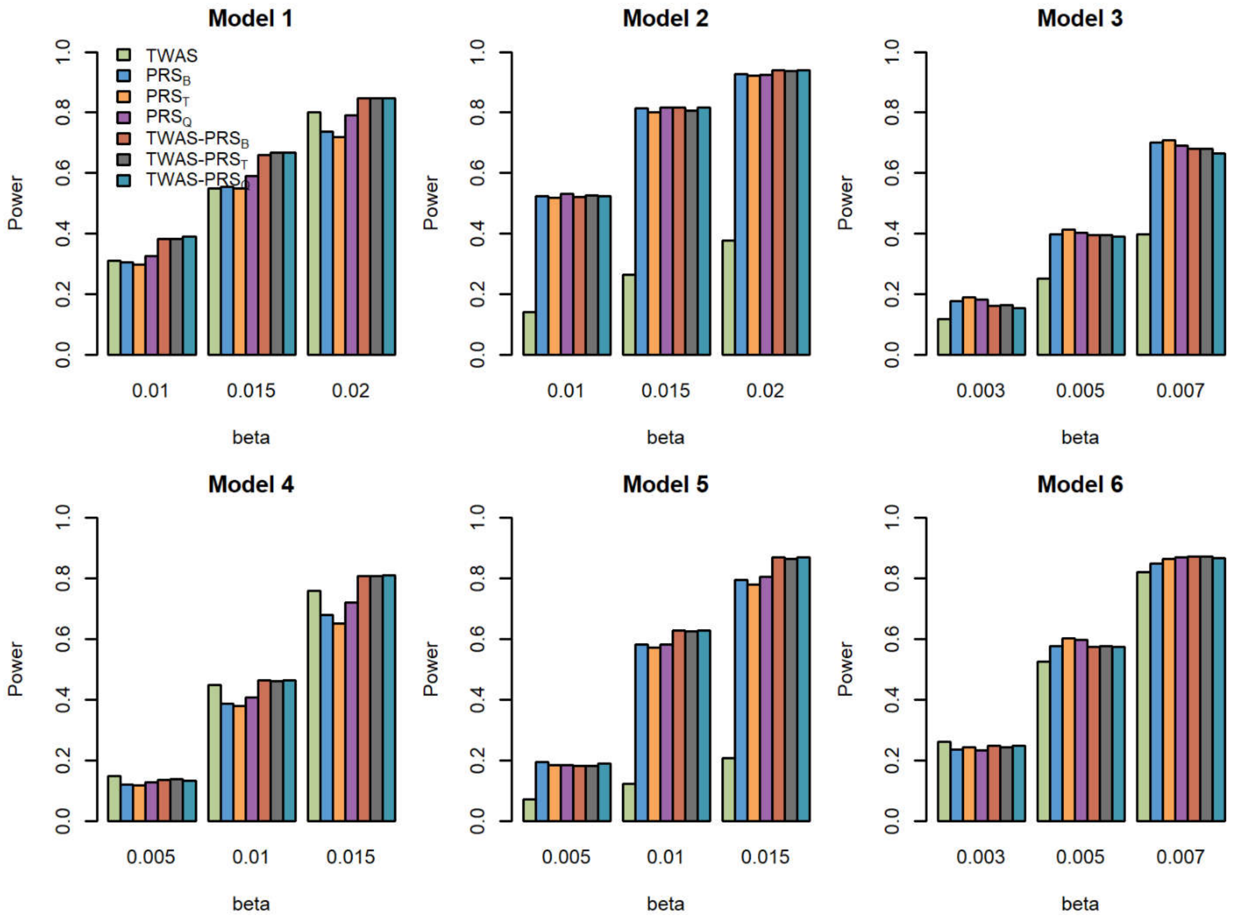

Figure S7. Powers of the seven tests versus the total effect size β for a qualitative trait with $N = 5,000$. The proportion of causal variants is 0.3. Models 1-3 corresponding to Genes 1-3 where we only use the eQTL with the largest weight to generate gene expression; Models 4-6 corresponding to Genes 1-3 where we use two eQTLs with the first two largest weights to generate gene expression.

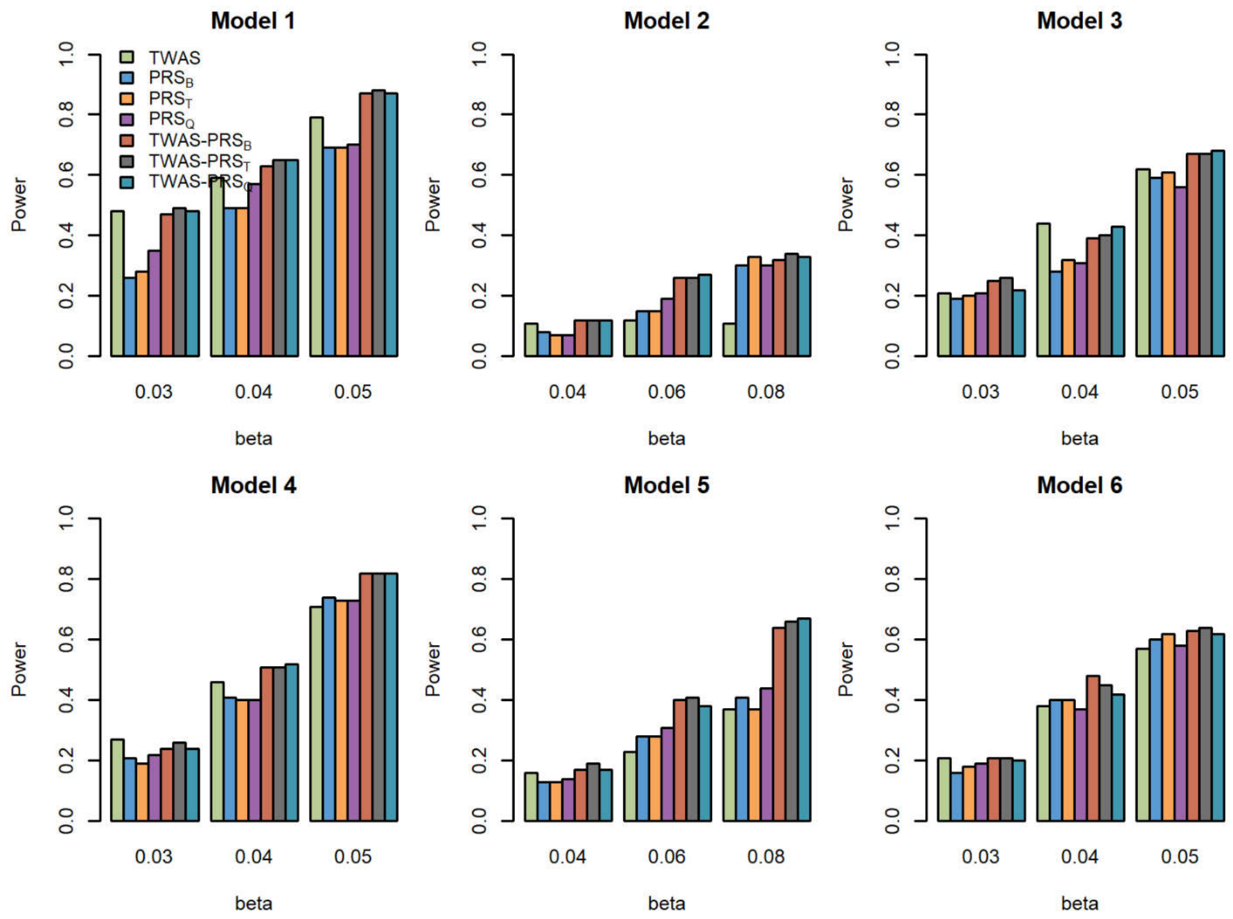

Figure S8. Powers of the seven tests versus the total effect size β for a qualitative trait with $N = 10,000$. The proportion of causal variants is 0.3. Models 1-3 corresponding to Genes 1-3 where we only use the eQTL with the largest weight to generate gene expression; Models 4-6 corresponding to Genes 1-3 where we use two eQTLs with the first two largest weights to generate gene expression.

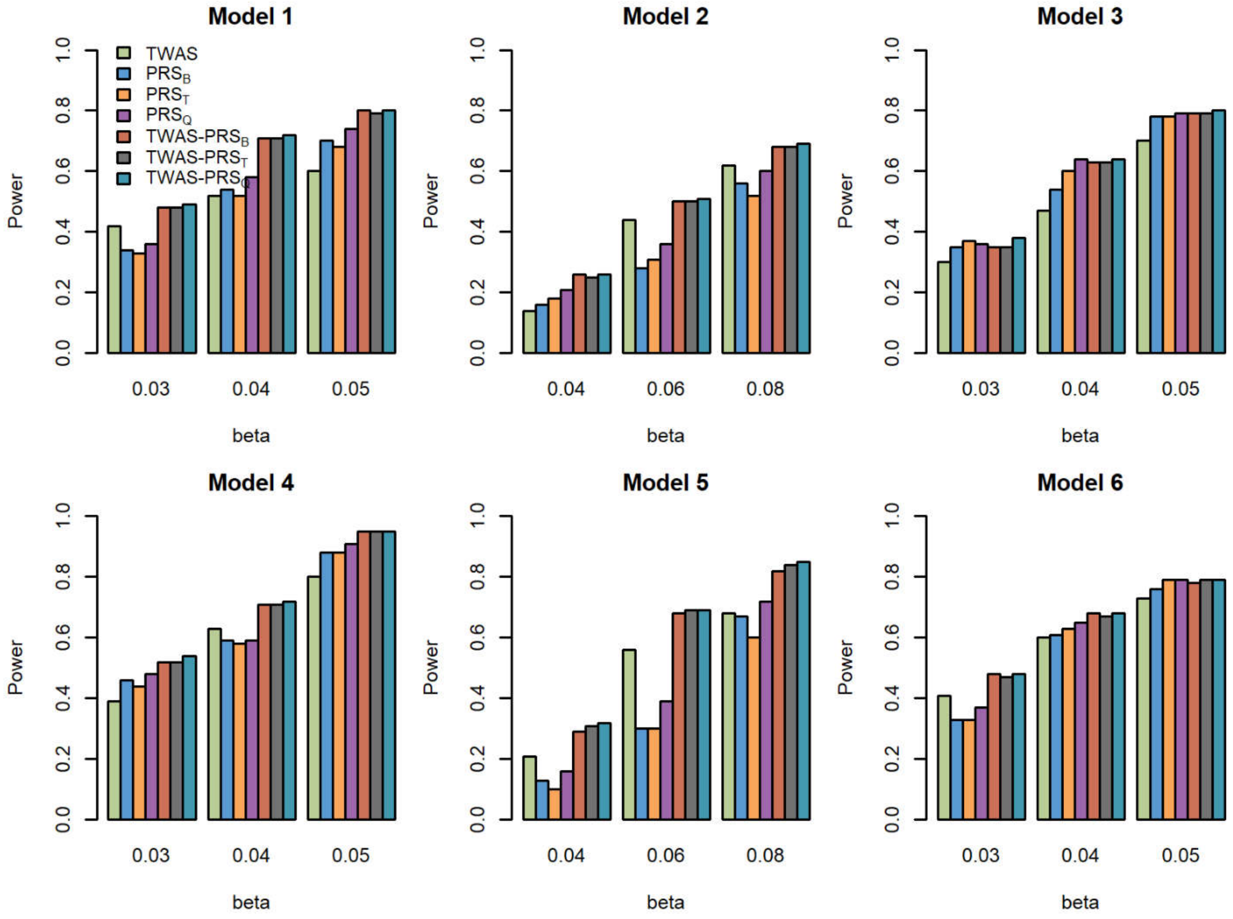

Figure S9. Powers of the seven tests versus the total effect size β for a qualitative trait with $N = 20,000$. The proportion of causal variants is 0.3. Models 1-3 corresponding to Genes 1-3 where we only use the eQTL with the largest weight to generate gene expression; Models 4-6 corresponding to Genes 1-3 where we use two eQTLs with the first two largest weights to generate gene expression.

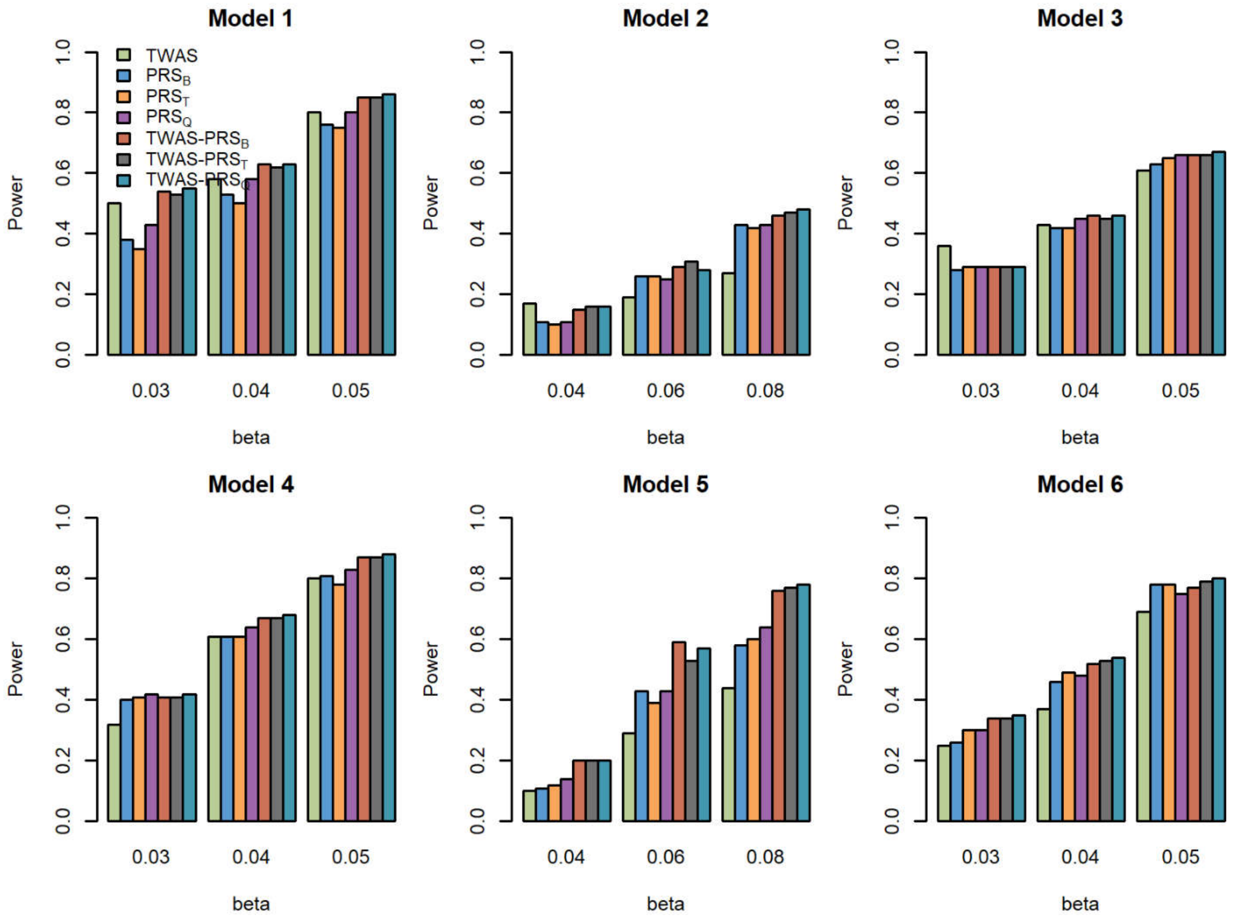

Supplement: Supplementary file 1 [file genes-13-01120-s001.zip › genes-1773975-supplementary.pdf]
